# (*E*)-Methyl 3-(1*H*-indol-2-yl)acrylate

**DOI:** 10.1107/S1600536811030121

**Published:** 2011-07-30

**Authors:** Rui-Bin Hou, Dong-Feng Li

**Affiliations:** a School of Chemistry and Life Sciences, Changchun University of Technology, Changchun 130012, People’s Republic of China

## Abstract

The title compound, C_12_H_11_NO_2_, is close to being planar (r.m.s. deviation for the non-H atoms = 0.033 Å). In the crystal, mol­ecules are linked by N—H⋯O hydrogen bonds, generating *C*(7) chains running along the *b* axis. A weak C—H⋯O interaction helps to establish the packing.

## Related literature

For background literature related to indoles in medicinal chemistry, see: Zeynep *et al.* (2005 ▶). For details of the synthesis, see García-Rubia *et al.* (2010 ▶).
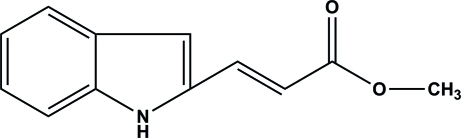


## Experimental

### 

#### Crystal data

C_12_H_11_NO_2_
*M* *_r_* = 201.22
Orthorhombic, 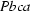

*a* = 7.735 (5) Å
*b* = 11.324 (5) Å
*c* = 23.236 (10) Å
*V* = 2035.4 (17) Å^3^
*Z* = 8
Mo *K*α radiation
μ = 0.09 mm^−1^
*T* = 296 K
0.39 × 0.27 × 0.22 mm

#### Data collection

Rigaku R-AXIS RAPID diffractometer
Absorption correction: multi-scan (*ABSCOR*; Higashi, 1995 ▶) *T*_min_ = 0.966, *T*_max_ = 0.980
18445 measured reflections
2326 independent reflections
1555 reflections with *I* > 2σ(*I*)
*R*_int_ = 0.065

#### Refinement

*R*[*F* ^2^ > 2σ(*F* ^2^)] = 0.049
*wR*(*F* ^2^) = 0.133
*S* = 1.04
2326 reflections
137 parameters
1 restraint
H-atom parameters constrained
Δρ_max_ = 0.14 e Å^−3^
Δρ_min_ = −0.19 e Å^−3^

### 

Data collection: *RAPID-AUTO* (Rigaku, 1998 ▶); cell refinement: *RAPID-AUTO*; data reduction: *CrystalStructure* (Rigaku/MSC, 2002 ▶); program(s) used to solve structure: *SHELXS97* (Sheldrick, 2008 ▶); program(s) used to refine structure: *SHELXL97* (Sheldrick, 2008 ▶); molecular graphics: *SHELXTL* (Sheldrick, 2008 ▶); software used to prepare material for publication: *SHELXL97*.

## Supplementary Material

Crystal structure: contains datablock(s) global, I. DOI: 10.1107/S1600536811030121/hb5951sup1.cif

Structure factors: contains datablock(s) I. DOI: 10.1107/S1600536811030121/hb5951Isup2.hkl

Supplementary material file. DOI: 10.1107/S1600536811030121/hb5951Isup3.cml

Additional supplementary materials:  crystallographic information; 3D view; checkCIF report

## Figures and Tables

**Table 1:** Hydrogen-bond geometry (Å, °)

*D*—H⋯*A*	*D*—H	H⋯*A*	*D*⋯*A*	*D*—H⋯*A*
N1—H1⋯O1^i^	0.98	1.93	2.900 (2)	174
C5—H5⋯O2^ii^	0.93	2.57	3.390 (3)	147

Symmetry codes: (i) 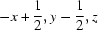; (ii) 
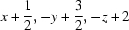
.

**Fig. 1.**
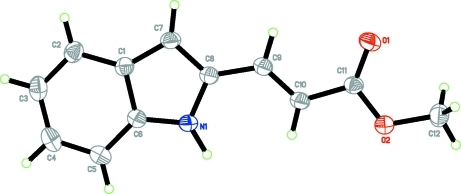
The title compound, with displacement ellipsoids of non-H atoms drawn at the 30% probalility level.

## supplementary crystallographic information

### Comment

Indole derivatives constitute an important class of therapeutic agents in medicinal chemistry including anticancer, antioxidant, antirheumatoidal and anti-HIVv (e.g. Zeynep *et al.*, 2005). We recently synthesized some indole derivatives as histone deacetylase (HDAC) inhibitors with the precursor. In this paper, we report the crystal structure of the title compound, (I).

The molecular structure of title compound, C_12_H_13_O_2_N, as shown in Fig. 1, all bond lengths and angles are in the normal ranges. All non-hydrogen atoms are nearly coplanar. In the crystal, the intermolecular N—H···O hydrogen bonds link the molecules into chains along b direction.

### Experimental

The title compound was prepared according to the literature method (García-Rubia *et al.*, 2010). Colourless blocks of (I) were prepared by slow evaporation of a solution in a mixture of dichloromethane and petroleum (60–90 °C) at room temperature.

### Refinement

Carbon-bound H-atoms were placed in calculated positions (C—H 0.93 and 0.97 Å) and were included in the refinement in the riding model with *U*_iso_(H) = 1.5 or 1.2 *U*_eq_(C). The N-bound H atom was located from a difference map and refined with the distance restraints N—H = 0.90 Å and *U*_iso_(H) = 1.5 *U*_eq_(N).

### Crystal data

**Table:**

C_12_H_11_NO_2_	*F*(000) = 848
*M**_r_* = 201.22	*D*_x_ = 1.313 Mg m^−^^3^
Orthorhombic, *P**b**c**a*	Mo *K*α radiation, λ = 0.71073 Å
Hall symbol: -P 2ac 2ab	Cell parameters from 10451 reflections
*a* = 7.735 (5) Å	θ = 3.2–27.5°
*b* = 11.324 (5) Å	µ = 0.09 mm^−^^1^
*c* = 23.236 (10) Å	*T* = 296 K
*V* = 2035.4 (17) Å^3^	Block, colorless
*Z* = 8	0.39 × 0.27 × 0.22 mm

### Data collection

**Table:**

Rigaku R-AXIS RAPID diffractometer	2326 independent reflections
Radiation source: fine-focus sealed tube	1555 reflections with *I* > 2σ(*I*)
graphite	*R*_int_ = 0.065
ω scans	θ_max_ = 27.5°, θ_min_ = 3.2°
Absorption correction: multi-scan (*ABSCOR*; Higashi, 1995)	*h* = −10→10
*T*_min_ = 0.966, *T*_max_ = 0.980	*k* = −14→13
18445 measured reflections	*l* = −30→29

### Refinement

**Table:**

Refinement on *F*^2^	Primary atom site location: structure-invariant direct methods
Least-squares matrix: full	Secondary atom site location: difference Fourier map
*R*[*F*^2^ > 2σ(*F*^2^)] = 0.049	Hydrogen site location: inferred from neighbouring sites
*wR*(*F*^2^) = 0.133	H-atom parameters constrained
*S* = 1.04	*w* = 1/[σ^2^(*F*_o_^2^) + (0.0668*P*)^2^ + 0.2072*P*] where *P* = (*F*_o_^2^ + 2*F*_c_^2^)/3
2326 reflections	(Δ/σ)_max_ = 0.003
137 parameters	Δρ_max_ = 0.14 e Å^−^^3^
1 restraint	Δρ_min_ = −0.19 e Å^−^^3^

### Special details

**Table:**

Experimental. (See detailed section in the paper)
Geometry. All e.s.d.'s (except the e.s.d. in the dihedral angle between two l.s. planes) are estimated using the full covariance matrix. The cell e.s.d.'s are taken into account individually in the estimation of e.s.d.'s in distances, angles and torsion angles; correlations between e.s.d.'s in cell parameters are only used when they are defined by crystal symmetry. An approximate (isotropic) treatment of cell e.s.d.'s is used for estimating e.s.d.'s involving l.s. planes.
Refinement. Refinement of *F*^2^ against ALL reflections. The weighted *R*-factor *wR* and goodness of fit *S* are based on *F*^2^, conventional *R*-factors *R* are based on *F*, with *F* set to zero for negative *F*^2^. The threshold expression of *F*^2^ > σ(*F*^2^) is used only for calculating *R*-factors(gt) *etc*. and is not relevant to the choice of reflections for refinement. *R*-factors based on *F*^2^ are statistically about twice as large as those based on *F*, and *R*- factors based on ALL data will be even larger.

### Fractional atomic coordinates and isotropic or equivalent isotropic displacement parameters (Å^2^)

**Table:**

	*x*	*y*	*z*	*U*_iso_*/*U*_eq_	
C1	0.5285 (2)	0.89360 (14)	0.82265 (7)	0.0413 (4)	
C2	0.6101 (3)	0.86472 (16)	0.77057 (7)	0.0505 (5)	
H2	0.6292	0.9224	0.7428	0.061*	
C3	0.6611 (3)	0.75051 (17)	0.76123 (8)	0.0571 (5)	
H3	0.7158	0.7310	0.7269	0.068*	
C4	0.6323 (3)	0.66264 (17)	0.80249 (9)	0.0592 (6)	
H4	0.6662	0.5855	0.7946	0.071*	
C5	0.5548 (3)	0.68769 (16)	0.85450 (8)	0.0511 (5)	
H5	0.5374	0.6293	0.8821	0.061*	
C6	0.5037 (2)	0.80400 (14)	0.86409 (7)	0.0399 (4)	
C7	0.4614 (2)	0.99847 (15)	0.84673 (7)	0.0448 (4)	
H7	0.4585	1.0721	0.8290	0.054*	
C8	0.4012 (2)	0.97292 (14)	0.90079 (7)	0.0401 (4)	
C9	0.3182 (2)	1.05130 (14)	0.94093 (7)	0.0421 (4)	
H9	0.3052	1.1292	0.9290	0.051*	
C10	0.2580 (2)	1.02608 (14)	0.99292 (7)	0.0422 (4)	
H10	0.2693	0.9498	1.0072	0.051*	
C11	0.1744 (2)	1.11639 (13)	1.02804 (7)	0.0397 (4)	
C12	0.0416 (3)	1.15364 (17)	1.11785 (8)	0.0573 (5)	
H12A	0.1057	1.2260	1.1206	0.086*	
H12B	0.0332	1.1180	1.1552	0.086*	
H12C	−0.0723	1.1699	1.1035	0.086*	
N1	0.4262 (2)	0.85400 (11)	0.91135 (6)	0.0409 (4)	
H1	0.3997	0.8130	0.9473	0.061*	
O1	0.1477 (2)	1.21728 (10)	1.01398 (5)	0.0635 (4)	
O2	0.12888 (17)	1.07412 (10)	1.07924 (5)	0.0484 (4)	

### Atomic displacement parameters (Å^2^)

**Table:**

	*U*^11^	*U*^22^	*U*^33^	*U*^12^	*U*^13^	*U*^23^
C1	0.0440 (11)	0.0430 (9)	0.0370 (8)	−0.0047 (7)	−0.0065 (7)	−0.0006 (7)
C2	0.0554 (13)	0.0565 (11)	0.0394 (9)	−0.0110 (9)	−0.0013 (8)	−0.0037 (8)
C3	0.0526 (13)	0.0653 (12)	0.0533 (11)	−0.0077 (10)	0.0060 (9)	−0.0191 (9)
C4	0.0546 (14)	0.0508 (11)	0.0724 (13)	0.0036 (9)	−0.0002 (10)	−0.0176 (10)
C5	0.0531 (12)	0.0391 (9)	0.0609 (11)	−0.0005 (8)	−0.0035 (9)	0.0007 (8)
C6	0.0383 (10)	0.0387 (8)	0.0428 (9)	−0.0013 (7)	−0.0046 (7)	−0.0006 (7)
C7	0.0548 (12)	0.0391 (8)	0.0405 (9)	−0.0009 (8)	−0.0044 (8)	0.0066 (7)
C8	0.0431 (10)	0.0365 (8)	0.0406 (9)	−0.0011 (7)	−0.0039 (7)	0.0014 (7)
C9	0.0462 (11)	0.0359 (8)	0.0443 (9)	0.0017 (7)	−0.0035 (8)	0.0016 (7)
C10	0.0475 (11)	0.0351 (8)	0.0440 (9)	0.0019 (7)	−0.0035 (8)	0.0037 (7)
C11	0.0442 (10)	0.0370 (9)	0.0377 (8)	−0.0009 (7)	−0.0048 (7)	0.0025 (7)
C12	0.0559 (13)	0.0626 (12)	0.0533 (11)	0.0067 (10)	0.0079 (9)	−0.0038 (9)
N1	0.0468 (9)	0.0362 (7)	0.0396 (7)	0.0000 (6)	0.0003 (6)	0.0048 (6)
O1	0.0983 (12)	0.0409 (7)	0.0514 (8)	0.0168 (7)	0.0050 (7)	0.0073 (6)
O2	0.0572 (9)	0.0428 (6)	0.0452 (7)	0.0047 (6)	0.0062 (6)	0.0043 (5)

### Geometric parameters (Å, °)

**Table:**

C1—C2	1.403 (2)	C8—N1	1.383 (2)
C1—C7	1.412 (2)	C8—C9	1.439 (2)
C1—C6	1.412 (2)	C9—C10	1.326 (2)
C2—C3	1.370 (3)	C9—H9	0.9300
C2—H2	0.9300	C10—C11	1.460 (2)
C3—C4	1.400 (3)	C10—H10	0.9300
C3—H3	0.9300	C11—O1	1.2060 (19)
C4—C5	1.378 (3)	C11—O2	1.330 (2)
C4—H4	0.9300	C12—O2	1.439 (2)
C5—C6	1.393 (2)	C12—H12A	0.9600
C5—H5	0.9300	C12—H12B	0.9600
C6—N1	1.373 (2)	C12—H12C	0.9600
C7—C8	1.371 (2)	N1—H1	0.9772
C7—H7	0.9300		
			
C2—C1—C7	134.69 (16)	C7—C8—C9	127.99 (15)
C2—C1—C6	118.79 (16)	N1—C8—C9	123.26 (15)
C7—C1—C6	106.51 (15)	C10—C9—C8	127.91 (16)
C3—C2—C1	119.11 (17)	C10—C9—H9	116.0
C3—C2—H2	120.4	C8—C9—H9	116.0
C1—C2—H2	120.4	C9—C10—C11	120.93 (15)
C2—C3—C4	121.12 (18)	C9—C10—H10	119.5
C2—C3—H3	119.4	C11—C10—H10	119.5
C4—C3—H3	119.4	O1—C11—O2	122.54 (15)
C5—C4—C3	121.56 (17)	O1—C11—C10	126.04 (15)
C5—C4—H4	119.2	O2—C11—C10	111.42 (13)
C3—C4—H4	119.2	O2—C12—H12A	109.5
C4—C5—C6	117.27 (17)	O2—C12—H12B	109.5
C4—C5—H5	121.4	H12A—C12—H12B	109.5
C6—C5—H5	121.4	O2—C12—H12C	109.5
N1—C6—C5	129.91 (16)	H12A—C12—H12C	109.5
N1—C6—C1	107.97 (14)	H12B—C12—H12C	109.5
C5—C6—C1	122.13 (16)	C6—N1—C8	108.71 (13)
C8—C7—C1	108.11 (15)	C6—N1—H1	125.4
C8—C7—H7	125.9	C8—N1—H1	125.8
C1—C7—H7	125.9	C11—O2—C12	117.17 (13)
C7—C8—N1	108.70 (15)		

### Hydrogen-bond geometry (Å, °)

**Table:**

*D*—H···*A*	*D*—H	H···*A*	*D*···*A*	*D*—H···*A*
N1—H1···O1^i^	0.98	1.93	2.900 (2)	174
C5—H5···O2^ii^	0.93	2.57	3.390 (3)	147

Symmetry codes: (i) −*x*+1/2, *y*−1/2, *z*; (ii) *x*+1/2, −*y*+3/2, −*z*+2.
